# Ecohydrology 2.0

**DOI:** 10.1007/s12210-022-01071-y

**Published:** 2022-05-04

**Authors:** Andrea Rinaldo, Ignacio Rodriguez-Iturbe

**Affiliations:** 1 Accademia Nazionale dei Lincei, Rome, Italy; 2 Laboratory of Ecohydrology ENAC/IIE/ECHO, École Polytechinque Fédérale de Lausanne, Lausanne, Switzerland; 3 Dipartimento ICEA, Università degli studi di Padova, Padua, Italy; 4 Department of Ocean Engineering, Texas A&M University, College Station, TX USA; 5 Department of Biological and Agricultural Engineering, Texas A&M University, College Station, TX USA

**Keywords:** General water controls on biota, Research hotspots, Biogeochemical cycles, Species populations pathogens catchment-scale control volumes, Rivers as ecological corridors

## Abstract

This paper aims at a definition of the domain of ecohydrology, a relatively new discipline borne out of an intrusion—as advertised by this Topical Collection of the Rendiconti Lincei—of hydrology and geomorphology into ecology (or vice-versa, depending on the reader’s background). The study of hydrologic controls on the biota proves, in our view, significantly broader than envisioned by its original focus that was centered on the critical zone where much of the action of soil, climate and vegetation interactions takes place. In this review of related topics and contributions, we propose a reasoned broadening of perspective, in particular by firmly centering ecohydrology on the fluvial catchment as its fundamental control volume. A substantial unity of materials and methods suggests that our advocacy may be considered legitimate.

## Introduction

Over 20 years ago, Peter S. Eagleson eloquently foresaw a new and challenging path for the hydrologic sciences (Eagleson 2000):

We need to get away from a view of hydrology as a purely physical science. Life on earth has to be a self-evident part of the discipline. In particular, I am thinking of vegetation, and its powerful interactive relationship with the atmosphere, at both a local and global scale. In attempting to get the full picture we must not be afraid to express the role of plants in our mathematical equations.

Similarly, around the same time one of us (Rodriguez-Iturbe 2000) defined ecohydrology as fundamentally concerned with:

[the] hydrologic mechanisms underlying the climate-soil-vegetation dynamics and thus controlling the most basic ecological patterns and processes.

The disciplinary reformulation of hydrology which the above implies was inserted in a new intellectual frontier for the environmental sciences. This new frontier, briefly described above, has been fundamentally widened in recent years. In ecohydrology we now focus not only in vegetation but in all aspects of life fundamentally related to hydrologic mechanisms. This new focus will transform our understanding of:

[..] basic processes that control the stability and sustainability of natural environmental systems. The ensuing findings will have extraordinary implications for our abilities to predict and manage how humans impact the health of ecosystems across local, regional, and global scales. Such knowledge is a critical component of a safe, sustainable, and prosperous future.

(quoted from L. Hedin et al. reporting to the US National Science Foundation Rodriguez-Iturbe 2005). From the wide landscape (*Hydrology and Life*), in this assessment we will focus on an important shift in the fundamental control volume of ecohydrologic analysis from the original focus on the critical zone (Grant and Dietrich 2017) (‘from bedrock to treetop’ to emphasize processes that manifest their spatial charactersmostly in the sub-vertical direction). The onset of a separate field of studies, methodologically and because of the breadth of the synthesis that proved necessary, was heralded by work on the probabilistic structure of soil-plant-atmosphere interactions (Rodriguez-Iturbe et al. 1999, 2009; Porporato and Yin 2022). Therein, the fundamental control volume is a unit area representative of at-a-point rainfall structure bounded by the earth surface on one hand and by the root zone depth on the other. Thus fluxes were essentially sub-vertical and storages respond to mass and momentum balance subsumed by constitutive equations based on solid experimental evidence from soil science and plant physiology. We advocate specific control volumes, of different nature, to define the domain of ecohydrology. Specifically, the fundamental control volumes of hydrology, the catchment, should instead be considered as the fundamental ecohydrological domain. The catchment brings to the fore much more complexity and many related applications (Rodriguez-Iturbe and Rinaldo 2001). It involves the properties of its embedded drainage network (a byproduct of the ontogenesis of the fluvial basin that generated the catchment delimitation) granting it unique controls and drivers over ecological process, well beyond the sole treatment of vegetation dynamics (Rinaldo et al. 2020). Thus, should we perhaps term *River Basins and Life* this endeavour, thereby extending the domain of interest of ecohydrology (Eagleson 2000; Rodriguez-Iturbe and Porporato 2004; Porporato and Yin 2022) from what is currently termed the critical zone (Grant and Dietrich 2017) also to the blended catchment, a system of hillslopes and channels making the catchment with all related characterizations e.g. (Montgomery and Dietrich 1988, 1992; Heimsath et al. 1997; Rodriguez-Iturbe and Rinaldo 2001). This is because river basins are geomorphological units of particular importance for life on earth and have been for years the focus of hydrologic and geomorphologic research (Rinaldo et al. 2020). Here, we will attempt to describe in a quantitative manner how the structure and organization of river basins are related to the stability and sustainability of the life they nourish. This extends, even for our teaching prospects (Porporato and Yin 2022), the study of water controls on biota, the truly federating feature of ecohydrological themes. Beyond the critical zone *sensu* (Grant and Dietrich 2017), *Ecohydrology 2.0* embeds mass and momentum balance equations of hydrologic fluxes and storages—quite possibly stochastic—not simply from *bedrock to treetop* to move on from soil-plant-atmosphere interactions at-a-point to embed the richness and complexity of water controls on species, populations and pathogens at the scale of whole catchments *sensu* (Rinaldo et al. 2020).

Merging problems and tools from ecology, hydrology, and geomorphology is, in our view, exciting and challenging. Currently, ecohydrologists mostly belong to the hydrology community, the largest self-declared share of current practitioners, and rarely to the ecologic community. They remain well separated academically, to the point that most ecologists refuse to survey relevant contributions published even in flagship hydrologic journals, whereas the same bias does not seem to affect hydrologists. Typically, even the term ecohydrology is currently chosen mostly by those whose origins are firmly in hydrology. A different issue is the relation with neighboring fields, like hydrobiology which is the brand name for an important interdisciplinary domain dealing with water—as opposed to hydrologic—controls of ecological and biological processes. Here the distinction appears reasonably clear, as hydrology pitches in when fluxes and storages need be estimated, rather than physical, chemical or biological properties related to the presence of water. We hope to somewhat contribute (as posited by Rinaldo et al. 2020) to the creation of common grounds for a widespread accessibility and acknowledgement leading to concepts and tools of one field to be immediately accessible to the others.

The development of the many interfaces of ecohydrology took some time to develop. It is now clear, for example, that river networks and the landscapes they portend are fundamental to define uniquely, and universally at least in runoff-generating areas, the substrate available to ecological interactions within the catchment, including the connectivity of many ecosystems and coupled human-natural systems. Details matter. For example, one simply cannot assume that the relevant features of a dendrite remain unaffected by constraints like the space-filling characters that may (or may not) be imposed in draining a catchment (Maritan et al. 1996; Rodriguez-Iturbe and Rinaldo 2001; Rinaldo et al. 2014), nor assume that topological measures of trees are truly distinctive features (Kirchner 1993; Rinaldo et al. 1999), to mention two of the most common misconceptions one finds in experimental and theoretical work drawn from the ecological literature. Topological features of spanning trees are inevitable and undistinctive of otherwise broadly different dendrites. Significantly, however, the inevitable topology of loopless trees proves a fundamental property for predicting biological invasions, where the probability of bifurcations along the backbone of propagation near the leading edge of a travelling wave controls its celerity (Campos and Mendez 2005; Campos et al. 2006; Bertuzzo et al. 2007; Méndez et al. 2010; Rinaldo et al. 2020). As another example, rainfall in space and time possesses stochastic features that have been studied for decades e.g. (Rodriguez-Iturbe et al. 1987; Bras and Rodriguez-Iturbe 1994) and it is unforgivable for an ecologist to venture in the territory of rainfall-driven ecological processes without a proper knowledge basis of what had been done in hydrology on the subject. Counting on the significant robustness shown by certain ecological models (e.g. Muneepeerakul et al. 2007; Rodriguez-Iturbe et al. 2009; Terui et al. 2018; Bertuzzo et al. 2015; Terui et al. 2014) would not always save the day (Rinaldo et al. 2020). The fundamental result may be summarized by the punchline stating that “neutral pattern does not imply neutral process” (Purves and Pacala 2005; Purves and Turnbull 2010). Indeed the predictive power of the neutral model of biodiversity and biogeography (Hubbell 1979, 2001) proves fundamental as the directional dispersal implied by the river network acting as the substrate for ecological interactions (aka ecological corridors) grants a hitherto unthinkable robustness to patterns of biodiversity (Muneepeerakul et al. 2007, 2008a, 2019; Rinaldo et al. 2020). This fundamental result has been confirmed by laboratory experimentation with living communities where nothing is postulated (or *de facto* imposed) to be neutral-like (Carrara et al. 2012, 2014). Metapopulation studies (Hanski 1998, 1999; Hanski and Ovaskainen 2002) within a networked landscape gain therefore particular significance e.g. (Fagan 2002; Muneepeerakul et al. 2007, 2008a; Fagan et al. 2009; Poff and Ward 1989; Terui et al. 2018, 2014; Campbell Grant et al. 2010; Campbell Grant 2011a; Liao et al. 2018; Ma et al. 2020; Giezendanner et al. 2021).

The long march has initiated more than a decade ago, along whose path several inroads into seemingly unrelated, now acknowledged, common grounds have been found among the various disciplines (Rinaldo et al. 2020). The theoretical tools rapidly expanded to belong in disease and conservation ecology, epidemiology, biomathematics, environmental engineering, water resources planning including biodiversity conservation, planetary health, and to some extent to complex systems epitomized by a few relevant network problems, e.g. data and models of human mobility that mean so much to the spread of epidemics (Rinaldo et al. 2012; Wesolowski et al. 2013, 2014; Finger et al. 2016, 2018), and multiplex networks that may affect biological invasions (Mari et al. 2009; Bertuzzo et al. 2011; Rinaldo et al. 2020). Our line of argument thus suggest unequivocally that an integrated ecohydrological framework, focused on hydrologic controls on the biota, has recurrent features hinging on the spatially explicit description of the interactions imposed by a very particular, and quite deeply studied, substrate for ecological interactions: the dendritic ecological corridors weaved together by river networks. They are characterized by universal features regardless of climate, vegetation, geology or exposed lithology everywhere in nature (Rodriguez-Iturbe and Rinaldo 2001) that bear significant consequences on large-scale ecological functions (e.g. Rinaldo et al. 2020). Significant predictions thus began to surface to describe metapopulation persistence in fluvial ecosystems metacommunity predictions of fish diversity patterns in large river basins, geomorphic controls imposed by the fluvial landscape on elevational gradients of species’ richness, biological invasions of very large and articulate river systems (Fagan 2002; Grant et al. 2007; Muneepeerakul et al. 2007; Economo and Keitt 2008; Muneepeerakul et al. 2008a; Economo and Keitt 2010; Campbell Grant et al. 2010; Goldberg et al. 2010; Bertuzzo et al. 2011; Campbell Grant 2011a; Mari et al. 2014; Zeigler and Fagan 2014; Muneepeerakul et al. 2019; Stoffels et al. 2016; Zhou and Fagan 2017; Liao et al. 2018; Ma et al. 2020; Giezendanner et al. 2021). The very same tools allowed us the reach out for addressing the spread of water-borne and water based diseases, whether endemic of epidemic. For example, predicting cholera epidemics, or range expansions of the incidence of endemic debilitating disease like schistosomiasis as a direct consequence of water resources management, now indeed within reach, stands as a symbol of our responsibilities for a fair distribution of water.

Also, new frontiers opened up for epidemiological and anthropological studies for which big data are now available: for example, by studies on the spread of proliferative kidney disease in salmonid fish for the formers (Carraro et al. 2016, 2017, 2018a, b), and for human mobility for the latters e.g. (Wesolowski et al. 2012, 2013; Finger et al. 2016). We now advocate for what we consider a compelling argument: ecological processes occurring in a catchment control volume are so constrained by hydrologic controls and by the morphology of the matrix for ecological interactions (e.g. the directional dispersal embedded in fluvial and mobility networks, host/pathogen relations for disease transmission, the features of the biological invasions) that the spatial and temporal patterns in ecology are indelibly marked by them. In some relevant cases, river networks are templates of such patterns (Rinaldo et al. 2020). In brief, it is all reflected in water. We thus propose to look beyond the current perimeter of the parent cultural origins, in a contamination process weaving together novel investigations on biodiversity and conservation.

In what follows we shall provide our view on research avenues that we believe are bound to soon become mainstream ecohydrology and in need for a broad knowledge of both ancestor fields, that is, an organic body of material ready for being taught and applied.

## Ecohydrology and the critical zone

This is the traditional domain of ecohydrology. We shall not dedicate in this context much space to it owing to its established nature, by now codified in monographs and textbooks (Rodriguez-Iturbe and Porporato 2004; Porporato and Yin 2022). In what follows we shall recall the main subjects where substantial progress has been achieved since the onset of the discipline (Rodriguez-Iturbe et al. 1999; Rodriguez-Iturbe 2000; D’Odorico et al. 2007; Porporato et al. 2002; D’Odorico et al. 2010, 2019).

The initial core business of ecohydrology was in soil and vegetation interactions mediated by hydrologic drivers and controls. Purportedly (Porporato and Yin 2022):

Ecohydrology is a fast-growing branch of science at the interface of ecology and geophysics, studying the interaction between soil, water, vegetation, microbiome, atmosphere, climate, and human society. This textbook gathers together the fundamentals of hydrology, ecology, environmental engineering, agronomy, and atmospheric science to provide a rigorous yet accessible description of the tools necessary for the mathematical modeling of water, energy, carbon, and nutrient transport within the soil–plant–atmosphere continuum. By focusing on the dynamics at multiple time scales, from the diurnal scale in the soil–plant–atmospheric system to the long-term stochastic dynamics of water availability, which is responsible for ecological patterns and environmental fluctuations, the book explains the impact of hydroclimatic variability on vegetation and soil microbial systems through biogeochemical cycles and ecosystems, under different socioeconomical pressures. It is aimed at advanced students, researchers, and professionals in hydrology, ecology, earth science, environmental engineering, environmental science, agronomy, and atmospheric science.

These aims are very much in line with those of the book’s ancestors (Eagleson 2000; Rodriguez-Iturbe and Porporato 2004; D’Odorico et al. 2019). In brief, the domain is referred to the dynamics of life and water within the critical zone.

Many fundamental achievements blessed the nascent field, in particular in the stochastic soil moisture dynamics leading to an exact characterization of the probabilistic modelling of water balance at a point, where the individual role and the mutual connections of climate, soil and vegetation were addressed (Rodriguez-Iturbe et al. 1999, 2001; Laio et al. 2001; Porporato et al. 2001). From the stochastic soil moisture dynamics, the related progress progressively moved from plant physiology and the nature of water stress to the shaping of ecosystems and soil carbon nitrogen cycles.

For a state-of-the-art assessment of the developments of the initial focus, the reader is referred to (Porporato and Yin 2022). In what follows, we discuss only a few examples because they are not seen as mainstream part of the discipline. We deem them particularly significant methodologically and relevant to science and society.

### The ecohydrology of hydroperiods and vegetation zonation

Looking across a tidal landscape, can one foretell signs of impending shifts, possibly catastrophic, among different possible geomorphological structures? Can one sort out the intertwined roles of ecology and hydrology in the interpretation of evident patterns of halophytic vegetation or marks of vegetation as ecosystem engineer of marsh morphology in estuarine, coastal or lagoonal waters? These are sample questions (Silvestri et al. 2005; Fagherazzi et al. 2006; Marani et al. 2007, 2013) of great ecological, cultural and socio-economic importance for tidal environments and their worldwide decline (Silvestri et al. 2005; Kirwan et al. 2007; Kirwan and Megonigal 2013). They also serve well the current efforts striving to define a coherent domain of ecohydrology. Within the domain of ecohydrology and the critical zone, that is, processes within basically a 0-dimensional spatial domain, the coupled tidal physical and biological processes belong rather naturally because the main driver of vegetation survival—and thus the inherent ability of tidal landforms to resist hydrodynamic demolition owing to the role of thriving halophytic vegetation—is the temporal extent of tidal flooding, compared to a relevant timescale determining the survival capabilities of the vegetation in the presence of oxygen depletion in the root zone.

In all the examples mentioned above, indeed a small view on a thriving field, it is clear that multiple equilibria, and transitions among them, appear in the evolutionary dynamics of tidal landforms. Vegetation type, disturbances of the benthic biofilm, sediment availability and marine transgressions or regressions drive the bio-geomorphic evolution of the system. Water controls are explicit in the relative sea level and in the frequency and the duration of flooding, whereas plant physiology determines the selective advantage of specific species of halophytes, and thus the ecology of zonation (Silvestri et al. 2005). Geomorphology pitches in because of the spatial role of accretion and deposition rates of organic and inorganic material shaping the topography of tidal landforms (D’Alpaos et al. 2005; Kirwan et al. 2007).

**Fig. 1:**
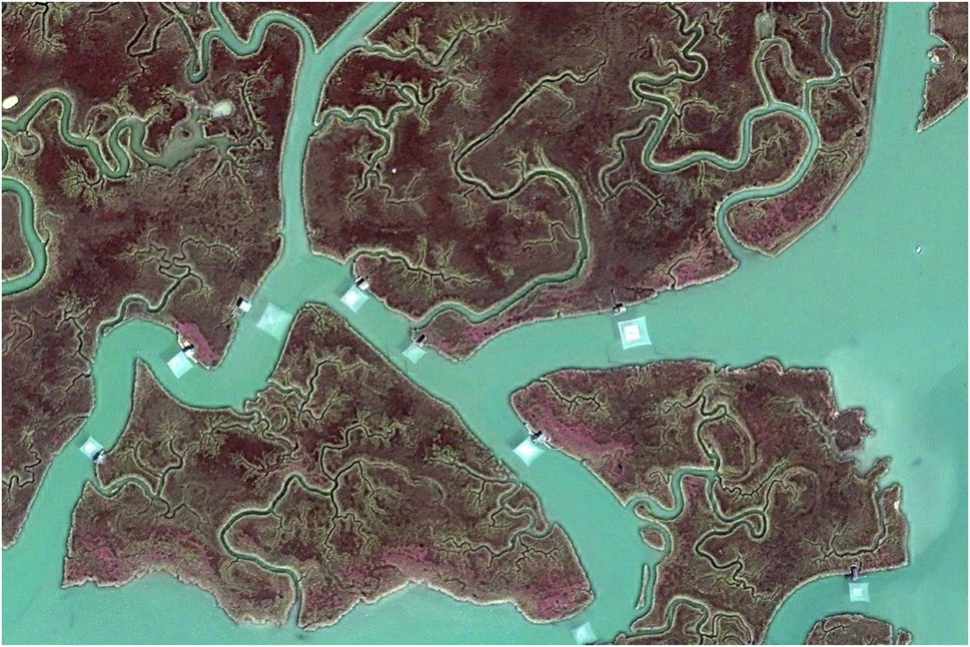
The beauty of the tidal landscape distracts from noting coexisting signatures of the built and the natural environments. The relevant morphodynamic processes exhibit significant complexity both in the planar organization and in the vertical direction, where accretion by organic and inorganic depositions contrast relative sea level. In this image, signs of the zonation of halophytic vegetation are shown. Zonation reflects precisely even minute differences in the saltmarsh topography regulating the tidal hydroperiod, in this case of astronomic origins, and thus precisely cycling, unavoidable and predictable tidal flooding aggravated by the combined effects of sea level rise and subsidence of the continental platform. The relevant selective pressure is the ability of the halophytes to withstand sustained oxygen depletion within the root zone. Suffocation due to excessive frequency of flooding and occupation of the ecological niche by a better competitor (after Marani et al. 2013). Anthropic effects here are reflected in the careful displacement of traditional fishing nets

Several approaches, some prone to significant exact results (Marani et al. 2007), provide general quantitative routes to model the fate of tidal landforms. A large body of empirical observations exists worldwide, at times spanning centuries, to fine tune the theoretical approaches. In some cases, theoretical approaches, combined with forecasts of relative sea-level, put forth scenarios predicting whether specific tidal landscapes and their saltmarshes, and their embedded ecosystem services, may survive or not. Preoccupations related to climatic changes are justified, even as soon as during the next century, if current IPCC scenarios of high relative sea level rise will materialize (Tognin et al. 2021). Figure 1 illustrates one such environment, in this case one of the few residual tidal landforms within the lagoon of Venice.

### Shifting rainfall patterns, soil microbial communities and carbon storage in soils

A key challenge for contemporary ecohydrology lies in the understanding of how different ecosystems respond to shifting water controls, and in identifying how hydrologic and climatic drivers mold the selective pressures shaping the evolution of living communities (Rodriguez-Iturbe and Rinaldo 2001; Rinaldo et al. 2020; Porporato and Yin 2022). A research area across hydrology, soil science and environmental microbiology will prove central, in our view. It originated by the study of iron-reducing bacteria that are primary mediators of anaerobic carbon oxidation, in particular in tropical forest soils where rainfall gradients may span very large gradients. The abundant rainfall and high net primary productivity of tropical forests provide optimal soil habitat for iron-reducing and iron-oxidizing bacteria (Dubinski et al. 2010). Spatially and temporally dynamic soil reduction-oxidation (redox) potentials, defined as a measure of the local tendency to donate or acquire electrons from the embedded environment, suggest conditions that make iron-transforming microbial communities central to the belowground carbon cycle in wet tropical forests, and, by natural extension, to global C-sinks (Peretyazkho and Sposito 2005; Bagnoud et al. 2016). Relatedly, soil heterotrophic respiration is the component of the terrestrial carbon cycle that determines whether soil ecosystems act as carbon sources or sinks. It has been recently established, on the basis of an analysis of a global database coupled to a probabilistic model of microbial growth, that the variability of ecosystem carbon source from microbial respiration is controlled by rainfall dynamics (Huang et al. 2021). Interestingly, the probability distribution of heterotrophic respiration proves to be controlled only by rainfall characteristics and vegetation productivity, thus positing that the propagation of fast hydrologic fluctuations prevail on the slower biological dynamics of microbial growth, and is remarkably independent of biome, soil type, and microbial physiology (Huang et al. 2021). This finding suggests that future changes in rainfall regime and net primary productivity may significantly, and predictably, alter the global soil carbon budget.

A fundamental role as driver of the evolution of soil microbial communities pertains the cycling of local redox potentials that underpin resource uptake and use by large and diverse soil microbial species assemblages. The role of redox cycling on the evolution of microbial communities and on carbon sinks in soils is fundamental (e.g. Peretyazkho and Sposito 2005; Silver et al. 1999; Magnabosco et al. 2018; Calabrese and Porporato 2019; Zhang and Furman 2021). In a nutshell, hydrologic stress is seen as a co-factor prompting fluctuations of the redox zone in soils, which is known to alter the composition of bacterial communities and their metabolic products including carbon sinks in soils (e.g. Richaume et al. 1989; Silver et al. 1999; Aller et al. 2010; Soucy et al. 2015; Rillig et al. 2017; Bagnoud et al. 2016). Necessary forthcoming ecohydrological interest needs therefore to blend, via both experiments and computations, the limits and the relevance of the role of hydrologic forcings subsumed by controlled irrigation and drainage regimes complemented by a proper characterization of relevant biogeochemical co-factors.

The importance of redox potential cycles can hardly be overestimated. In fact, highly dynamic redox conditions and fluctuations in organic carbon result in environmental conditions for microbial communities that require rapid adaptation. The faster the redox fluctuation, the more versatile microorganisms need to be to retain competitiveness. Thus soils become veritable evolutionary incubators because horizontal gene transfer (HGT) and point mutations prompt change in genetic material retained under the relevant selection pressure (Rillig et al. 2017). To quantify how different assemblages of communities are shaped, one needs to characterize the way in which natural and modified strains shape communities in a process where ecological and evolutionary timescales often prove comparable (Magnabosco et al. 2018). It seems that a significant task of ecohydrology is to address byproducts of metabolic activity operated by various assemblages of soil microorganisms under fluctuating, environmentally relevant conditions. This demands the determination of redox potentials in space and time as a prerequisite. The evolution of soil microbial communities is recapitulated by the cycling of redox potential, and thus first requires the (relatively accessible) determination of frequency, duration and fluctuations of soil saturation e.g. (Ebrahimi and Or 2015; Tecon and Or 2017; Borer et al. 2018). However, such a determination does not suffice. Specifically, ecohydrology needs to address how redox regimes vary when subjected to dynamic variations of soil saturation and changing carbon sources (Simkus 2016; Magnabosco et al. 2018), in particular when driven by shifts in the depth of soil saturation due to controlled irrigation and drainage regimes possibly in the presence of vegetation and complex root systems (e.g. Queloz et al. 2015; Benettin et al. 2021). Ecohydrology thus needs to address at a fundamental level the causes of composition and activity of the microbial community (Simkus 2016), and related community effects. One reason to expect these effects is the presence of biogeochemical and hydrologic gradients and their interdependences. Indeed, bacterial communities are key players in global biochemical cycles (Falkowski et al. 2008) as they regulate ecosystem functions in all of the biosphere (Gibbons and Gilbert 2015; Borer and Or 2021).

Microbial characterizations in a proper ecological and evolutionary perspective will soon come through extended and replicated experimentation. Creating experimental conditions that prompt the most rapid evolution remains a challenging task, however, as they require not only a meaningful description of the hydrologic stress prompted by sequences of oxic/anoxic conditions, but also a comprehensive view of different biogeochemical drivers affecting redox potential cycles. The central role of soil wetness in shaping bacterial abundance is nevertheless acknowledged (Bickel and Or 2021). However, the possible presence of exotic carbon sources, or the exposure to toxic metals may be significant to decisive co-factors (Bagnoud et al. 2016). Forthcoming work will likely concentrate on the predictability of the mechanisms by which redox cycling is generated thus potentially impacting microbial soil carbon utilization. The long term goal must be that of understanding: (i) shifts in the microbial community in response to cycling redox conditions favouring the growth of specific organisms able to utilize the available carbon; and (ii) the availability of specific electron acceptors depending on how the geochemical regimes influence the type of organic carbon that can be degraded. Relevant to our discussion is the fact that the sole hydrologic control does not suffice. In fact, fluid chemistry changes are one of the leading drivers of spatial and temporal distributions of surface and subsurface microbial communities and their activity. Continental scale estimations of cell distribution converges to a negative correlation of cell concentration versus depth (Magnabosco et al. 2018). In that pioneering work, coarse-grained estimates ranging over 3 or more orders of magnitude in the vertical direction are consistent with general trend of heat flow, hydrology, geology properties and surface temperature. However, they lump small fluctuations and fail to catch inverse trends arising in local spatial cell abundance as a function of nutrient availability (e.g. organic carbon concentrations). Cell presence and organic carbon concentrations positively correlate in soil zones up to the first few meters, and negatively correlate for deeper zones (Magnabosco et al. 2018). Average mobile-immobile ratios in groundwater cell concentration differs from the ones extrapolated by direct cell count-versus-deep relationships (Federle et al. 1986; Hazen et al. 1991), suggesting that microbial colonization in wet soils is a complex phenomenon still poorly understood. In this area we expect major ecohydrological contributions in the near future.

## Ecohydrology and the catchment scale

In our view, the domain of ecohydrology has been significantly expanded in the last decade to embed other hydrologic (and, relatedly, geomorphic) controls. Specifically, (Rinaldo et al. 2020), the field now:

$\ldots$ draws together several lines of argument to suggest that an integrated ecohydrological framework, which blends laboratory, field and theoretical evidence focused on hydrologic controls on biota, has contributed substantially to our understanding of the function of river networks as ecological corridors. This function is relevant to a number of key ecological processes. Jointly with other determinants will discuss, these processes control the spatial ecology of species and biodiversity in the river basin, the population dynamics and biological invasions along waterways, and the spread of waterborne disease. As revealing examples, here we describe metapopulation persistence in fluvial ecosystems, metacommunity predictions of fish diversity patterns in large river basins, geomorphic controls imposed by the fluvial landscape on elevational gradients of species’ richness, zebra mussel invasions of an iconic river network, and the spread of proliferative kidney disease in salmonid fish. Our main tenet is that ecological processes in the fluvial landscape are constrained by hydrology and by the matrix for ecological interactions (notably, the directional dispersal embedded in fluvial and host/pathogen mobility networks). Accounting for these drivers requires spatial descriptions that have now produced a remarkably broad range of results which are worth being recapped in a book illustrating the coherent framework that produced them. In brief, the overarching theme $\ldots$ is an investigation on how the physical structure of the environment affects biodiversity, species invasions, survival and extinction, and waterborne disease spread. Such a relation is explored from the (somewhat narrow, yet significant in our view) perspective of ecosystems produced by fluvial processes and forms whose origins and features we have studied for a long time.

In what follows, we provide a few examples of relevant research prospects that extend and complement the original domain of ecohydrological studies. The examples, by no means exhaustive, pertain what we believe will happen next in terms of relevant applications, a fitting task for the scopes of this paper.

### Metapopulation studies and the catchment

To capture the ecological dynamics of species constrained to disperse within river networks, a fundamental role is played by metapopulation dynamics (Hanski 1998, 1999; Hanski and Ovaskainen 2000; Hanski 2016), or the study of the probability of survival of a focus species regardless of interspecies’ interactions and solely subject to local colonization-extinction dynamics and regional dispersal ability (Campbell Grant et al. 2010; Fagan 2002; Muneepeerakul et al. 2008a). While the reader is referred to general sources for the many related contributions e.g. (Rinaldo et al. 2020), here we shall concentrate on the ecological consequences of the inherently scaling structure of the river system—a property that allows any reasonable model of species dynamics to predict species occupancy towards the upper reaches of the river and the suitability of dispersal strategies. Our take is that metapopulation persistence is non-trivially related to the structure of fluvial network (Mari et al. 2014; Giezendanner et al. 2021). Two examples of significant results follow.

Longitudinal gradients in width and depth of channel reaches depend on the very fabric of the river network: the spatial aggregation structure (Leopold et al. 1964; Benda et al. 2004) as the longitudinal addition of tributaries makes for predictable gains in runoff for landscape-forming events (Rodriguez-Iturbe and Rinaldo 2001). Fluvial landscapes are known to attain stationary network configurations that settle in dynamically accessible minima of total energy dissipation by landscape-forming discharges (Rodriguez-Iturbe and Rinaldo 2001; Rinaldo et al. 2014). The study of the connections between the recurrent features of the aggregation structure of a river system with the general viability of a dendritic substrate for ecological interactions has been first investigated by (Bertuzzo et al. 2015). The relation between minimization of total energy dissipation, the physical driver for the evolution of river networks, and the ecological dynamics of their embedded biota reveals interesting, not yet fully explained features. Sequences of OCN configurations are known to relate network selection to general landscape evolution equations (Banavar et al. 2001) through imperfect searches for dynamically accessible states (Rinaldo et al. 2014). The latters emerge from the selection process regardless of quenched randomness imposed on the dynamics to mimic processes frustrated by the vagaries of nature (Bak 1996). In particular, relations were sought among the processes that shape metric and topological properties of river networks, prescribed by physical constraints in landscape evolution, and those affecting the landscape capacity to support metapopulations—yet another view on biodiversity in fluvial ecosystems. Fluvial landforms show empirically (and compellingly) profound similarities of the parts and the whole across several orders of magnitude regardless of major diversities in their drivers and controls, like geology, exposed lithology, vegetation and climate (Rodriguez-Iturbe and Rinaldo 2001). River networks in runoff-generating areas are spanning trees (Banavar et al. 1997, 2000, 2001): a unique route exists for landscape-forming discharges from every site to an outlet, and no loops are observed. Optimal channel networks (OCNs) are trees minimizing a functional describing total energy dissipated along drainage directions by landscape-forming discharges which hierarchically accumulate towards the outlet of the basin. OCNs are exact steady-state solutions of the general landscape evolution equation under the small gradient approximation, and any loopless network configuration that minimizes total energy dissipation corresponds exactly to stationary solutions of the general landscape evolution equation under reparametrization invariance in the small-gradient approximation (Banavar et al. 2000, 2001; Rinaldo et al. 2006, 2014). The large variety of dynamically accessible local optima and the universality of their scaling features akin to those observed in nature suggested several applications ranging from the design of experiments in the laboratory e.g. Carrara et al. (2012, 2014) to a variety of explorations on network scaling (Briggs and Krishnamoorthy 2013) also prompted by the availability of computational tools for the generation of OCN trees and landscapes (Carraro et al. 2020b). The latters include, when theoretically feasible (Banavar et al. 2001; Balister et al. 2018), the nontrivial construction of three-dimensional self-affine landscapes from planar trees (Rodriguez-Iturbe et al. 1994; Rodriguez-Iturbe and Rinaldo 2001; Rinaldo et al. 2014).

Understanding the origins, and the needs for maintenance, of biodiversity in dendritic freshwater metacommunities is a primary goal of current ecological studies centered on population demography, population genetics and community composition. In this context, river networks are important testbeds, viewed as ecological corridors for species, populations and pathogens (Rinaldo et al. 2020). Interestingly, the theoretical prediction for a key role of dendritic connectivity in shaping biodiversity patterns resists several generalizations, from individual-based to metacommunity models and for interactions/migrations ranging from nearest neighbors alone to long distances (Muneepeerakul et al. 2007, 2008b; Rodriguez-Iturbe et al. 2009). Replicated experimental evidence that connectivity per se shapes diversity patterns in microcosm metacommunities supports such tenet (Carrara et al. 2012, 2014), jointly with abundant empirical evidence e.g. Fagan (2002), Benda (2004) and Campbell Grant (2011b). Spatially constrained dendritic connectivity is now accepted as a key factor for community composition and population persistence in environmental matrices commonly found in many natural and ecosystems, see e.g. Fraser et al. (2004), Haddad (1999), Markwith and Scanlon (2007) and Altermatt et al. (2011). Borrowing concepts from metapopulation ecology (Hanski 1998, 1999), landscapes may be viewed as networks of connected habitat patches in which species consist of local populations connected by dispersal or migration. A core topic of metapopulation ecology concerns the study of the conditions leading to regional persistence of species with fluctuating local populations. This is achieved by balancing the effects of basic rates characteristic of the population with the large-scale dynamic consequences of spatial effects (i.e. migrations and dispersal) involving local populations. This leads to the definition of metapopulation capacity (Hanski 1999) as an objective measure to link landscape structures with their capacity to sustain survival of a viable population of the focus species. Because metapopulation capacity can be conveniently used to rank different landscapes in terms of their capacity to support metapopulations, the study of how it changes in response to the evolving network configurations of spanning trees has interest.

**Fig. 2:**
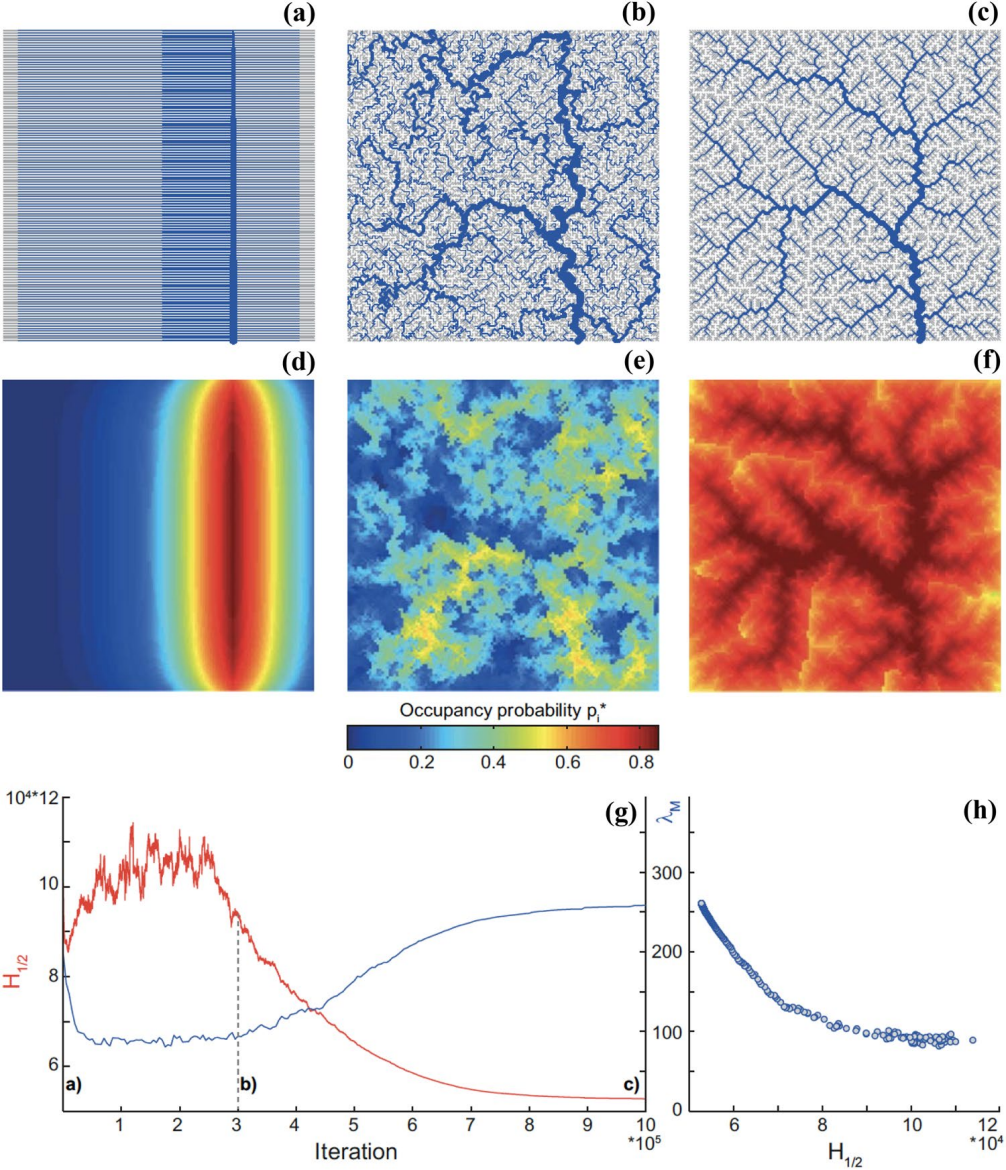
Unintended consequences: The process of minimizing total energy dissipation that leads to an Optimal Channel Network (OCN) (Rodriguez-Iturbe and Rinaldo 2001; Rinaldo et al. 2014) implies opposite changes in the metapopulation capacity of the embedded fluvial landscape: **a** initial network configuration, characterized by parallel flow directions collected by a central channel whose outlet is placed at the lower boundary; **b** intermediate state characterized by a disordered structure; and **c** final OCN. **d**–**f** Spatial distribution of the equilibrium probability of occupancy $p_i$ for the network configurations shown above; **g** evolution along the iteration of the simulated annealing process of total energy dissipation (red) and metapopulation capacity (blue). One thus verifies empirically **h** that energy minimization of a network configuration results in improved metapopulation capacities, here underpinned by the correlation between metapopulation capacities ($\lambda _M$) and evolving values of the functional ($H_{1/2}$) defining total energy dissipation (after Bertuzzo et al. 2015) (color figure online)

Figure 2 shows the results of a computational experiment carried out on a large lattice starting from an initial condition characterized by parallel flow channels draining onto an orthogonal collecting channel (Fig. 2a). The whole process of optimization of the energy functional ($H_{1/2}$) is illustrated here, including the initial phase where changes were accepted even if the related energy increased to reduce the imprinting of the initial condition (Rodriguez-Iturbe and Rinaldo 2001). Figure 2b shows an intermediate configuration along the minimum search process. Figure 2c shows the network obtained after the algorithm converged towards a local minimum of energy dissipation, showing features indistinguishable from natural forms unlike chance-dominated constructs (Rodriguez-Iturbe and Rinaldo 2001; Rinaldo et al. 1999). Interestingly, along the process of minimizing total energy dissipation by shifting and sorting landscape matrices, the metapopulation capacity of the resulting landscapes increases. The evolution of $\lambda _M$ mirrors, with opposite sign, the convergence of total energy dissipation towards its local minimum. Figure 2 also shows the spatial distribution of the equilibrium occupancy probability of the population of a focus species spreading in the corresponding networks. Note that the chosen intermediate state in the process of optimization, lowering only marginally the initial energy value, is characterized by a disordered aggregation process far from locally optimal either in terms of energy dissipation or in terms of metapopulation capacity. The occupancy probability of the OCN is instead characterized by vastly improved values indicating higher chances of species persistence and occupancy. Another pattern is clearly distinguishable in Fig. 2, occupancy probability increases moving from headwaters downstream, although with a final decrease towards the outlet of the catchment.

Figure 2 suggests that minimizing energy may increase metapopulation capacity. One thus wonders whether a local minimum of total energy dissipation, prescribed by the OCN search, corresponds to a local maximum of $\lambda _M$. This turns out to be not the case (Bertuzzo et al. 2015). This implies different distributions of the occupancy probability with higher mean, that is, landscapes more supportive for metapopulations to persist. Crucially, it was shown therein (Bertuzzo et al. 2015) that the aggregation structures produced by maximization of metapopulation capacity are very different from the forms that we observe in nature for river networks, nor they imply lower total energy dissipation. In any case, it must be remarked that Darwinian evolution (and the associated optimality criteria which have been the object of lively discussions in the past century) does not at all necessarily imply the maximization of metapopulation capacity, although this kind of maximization might vaguely remind the so-called *K*-selection (Pianka 1970), which implies maximization of the species carrying capacity. In any case, unintended ecological consequences of physical constraints are a worth research subject for future investigations.

The second example of metapopulation studies epitomizes the connections of hydrology, geomorphology and ecology and concerns the time variability of species viability within time-changing waterscapes (e.g. Malcolm et al. 2007a, b; Stoffels et al. 2016; Zhou and Fagan 2017). Specifically, (Giezendanner et al. 2021) wondered whether a dynamic drainage density of flowing fluvial networks may have a direct and predictable role on species persistence. Surely, the general viability of a focus species relevant to streamflow ecology depends on hydrologic factors that change predictably within the river network and on ecological processes integrating what happens in the upstream catchment. Despite the evidence of the major biogeochemical and hydrologic relevance of the fluctuations of the flowing extent of a river network (Battin et al. 2009; Basu et al. 2011; Bertuzzo et al. 2011; Godsey and Kirchner 2014; Ceola et al. 2013, 2014; van Meerveld et al. 2019; Botter and Durighetto 2020), their ecological implications have not been fully clarified. Scaling tree-like nested structures (Rodriguez-Iturbe and Rinaldo 2001) posit that local storages and fluxes depend on aggregation, thus bringing fundamental nonlocal interactions into the ecological dynamics (see e.g. Bertuzzo et al. 2011; Rinaldo et al. 2020). In this context, the description of temporary and ephemeral streams, although certainly not new (Gregory and Walling 1968; Day 1978; Bernier 1985), is currently a major focus of ecohydrologic research where field studies aim at quantifying the seasonal and event-based dynamics of the active stream network and their implications (Jaeger et al. 2007; Agren et al. 2015; Shaw 2016; Whiting and Godsey 2016; Perez-Saez et al. 2016, 2017; Lovill et al. 2018; Floriancic et al. 2018; Perez-Saez et al. 2019; van Meerveld et al. 2019; Durighetto et al. 2020).

Transient waterscapes and their connectivity have been the subject of many ecological investigations (e.g. Unmack 2001; Dixon 2003; Trexler et al. 2005; Tetzlaff et al. 2007; De Angelis et al. 2005; Zeigler and Fagan 2014; Stoffels et al. 2016; Zhou and Fagan 2017; Bertassello et al. 2019; Lowe et al. 2019; Bertassello et al. 2020a, b, 2022). Changes in size, shape and connection of habitat patches induced by water level rises may generate ecological opportunities due to transient windows for connectivity, periodic or erratic (Lodge 2004; Zeigler and Fagan 2014; Bertassello et al. 2019, 2022). Short-lived connectivities may be exploited by fish (Zeigler and Fagan 2014) or amphibians (Bertassello et al. 2019; Lowe et al. 2019) also in relation to the habitat size offered by the permanent water body (e.g. Rinaldo et al. 2020). Seasonal fluctuations may affect also terrestrial species, in particular riparian tree species whose recruitment and establishment need suitable seed transport and depositional environments (Dixon 2003). Related issues concern the strategies for species survival in ephemeral rivers e.g. via aestivation (Kerezst et al. 2013; Perez-Saez et al. 2019) and the spatial variation in the size of the fluvial branches in promoting metapopulation persistence in river networks (Ma et al. 2020).

The broad characters of the persistence of specific species to spatially and temporally varying hydrologic connectivity depend on factors centered on the interplay between their dispersal ability and the extent of the fluctuations of the habitat size and its inherent risks (Unmack 2001; Mari et al. 2014; Stoffels et al. 2016). Chief among the latters, e.g. for small fish, is the risk of getting trapped in isolated patches generated during the retreating phase to the permanent water bodies, that may eventually dry out causing periodic bouts of mortality. Rivers impose significant constraints to aquatic organisms, like those induced by a strong downstream drift or minimum stage thresholds that might prevent fish migration (e.g. Tetzlaff et al. 2018; Gonzáles-Ferreira et al. 2019). Plants and animals may persist in fluvial ecosystems however, which actually harbor great biological diversity. All these factors act synergistically with fluvial geomorphology, which is characterized by hierarchical branching geometries and universal scaling features of their master variables of aggregation and length (Rodriguez-Iturbe and Rinaldo 2001), and stream ecosystems whose branched structure is an important constraining factor for aquatic species that lack life stages that can disperse overland (Fagan 2002; Zeigler and Fagan 2014).

**Fig. 3:**
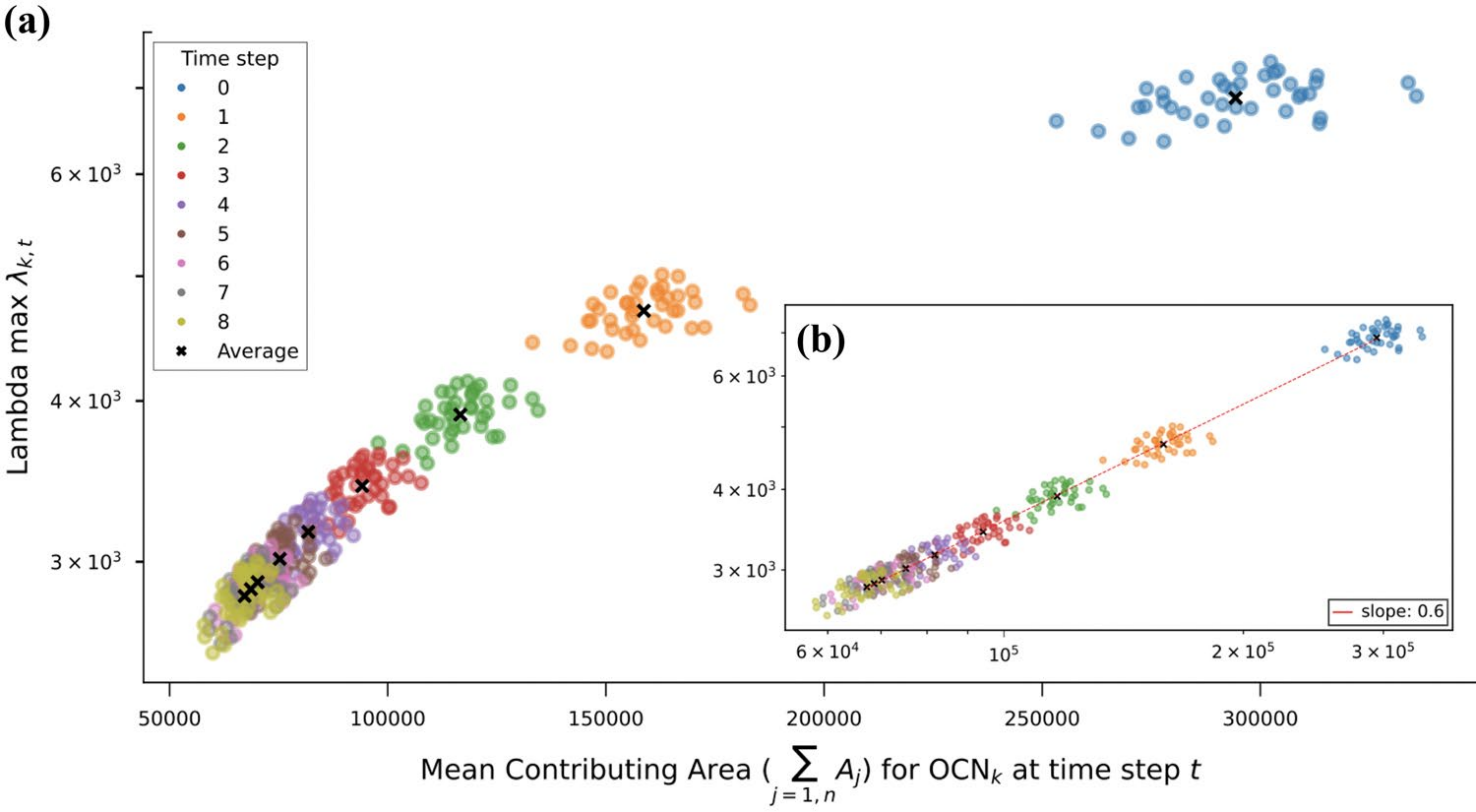
Scaling of species viability with time-varying geomorphic measures: **a** mean active contributing area ${\bar{A}} = 1/n \sum _{j=1,n} A_j$ of the flowing river network at various stages *t* of the network retraction, plotted against metapopulation capacity (Hanski 1998, 1999) $\lambda _{k,t}$ for each *k*-th replica optimal channel network (Rodriguez-Iturbe and Rinaldo 2001) at various stages *t* of seasonal contraction/expansion. The *y*-axis is log-scaled. Inset **b**: same as **a** in a log-log plot. The relation between the mean data (dashed line) proves concave in log-space with slope $\sim 0.6 \pm 0.05$ (after Giezendanner et al. 2021)

A theoretical exercise (Giezendanner et al. 2021), its limitations notwithstanding, subsumes the way forward in this field. Figure 3 shows scaling of species viability—again recapitulated by the substrate’s metapopulation capacity (Hanski and Ovaskainen 2000)—with time-varying geomorphic measures of an idealized, replicated spanning river network periodically and seasonally contracting and expanding. Figure 3 shows the scaling relation between the chosen measure of species viability with a meaningful time-dependent geomorphic measure. The measure of species viability is the metapopulation capacity (Hanski 1998, 1999; Hanski and Ovaskainen 2000) of the dendritic fluvial domain, $\lambda _{k,t}$, where the index *k* refers to one of the independent statistical replicas of a spanning optimal channel network (Rodriguez-Iturbe and Rinaldo 2001) grown in the same domain, and *t* indexes the various stages *t* of seasonal contractions or expansions of the flowing fluvial domain. The geomorphic measure that defines viability is the mean active contributing area ${\bar{A}} = 1/n \sum _{j=1,n} A_j$ of the flowing river network, where *n* is the total number of sites and $A_j$ is the unique value of total contributing area at any site *j*, which in turn is proportional to the mean network length (Giezendanner et al. 2021).

The empirical verification that the variation of the number of sites (“patches”) within a network, and the variation in the total amount of habitat (seen as the total pooled area of active pixels) do not explain but a fraction of the actually occupied sites averaged in time (Hanski et al. 2015) supports the theoretical result highlighted in Fig. 3. Metapopulation theory suggests that the equilibrium value of the probability of occupancy of the *i*-th site, say $p_i$, is a weighted average of patch occupancy where the weights describe the role of individual sites in the dynamics of the metapopulation (Hanski and Ovaskainen 2000). The equilibrium value $p_i$, the onset of persistence, is shown to be equal to $p_i = 1 - \frac{e/c}{\lambda _M}$, where $\lambda _M$ is a suitable average of the metapopulation capacities $\lambda _{(k,t)}$ of single realizations under the assumptions of the model (Giezendanner et al. 2021). The ratio *e*/*c* characterizes the focus species via the extinction and colonization rates (Hanski and Ovaskainen 2000) highlighting that the extinction threshold is a characteristic of the focus species, whereas the metapopulation capacity describes the features of the connected ecological substrate network—the relative importance of dispersal rates and the relative distance among all patches is contained in the metapopulation capacity—being the maximum eigenvalue of a landscape matrix (Hanski and Ovaskainen 2000). The result in Fig. 3 suggests that, should empirical validations support a power-law relation between metapopulation capacity and the network diameter (the mean distance between any two sites of the flowing network) subsumed by the mean total contributing area i.e. $\lambda _M \sim \mathcal{{K}} {\bar{L}}^{0.6}$ (where $\mathcal{{K}}$ is a proportionality constant that may be estimated from the data in Fig. 3), a relation of the type

1 $$\begin{aligned} p_i = 1 - \frac{e/c}{ \mathcal{{K}} {\bar{L}}^{0.6}} \end{aligned}$$

where $\lambda _M$ is replaced by the ensemble mean and time averaged value metapopulation capacities. Such a relation could be validated by empirical studies if $p_i$ would be measured accurately enough as the fraction of times in which patch *i* has been occupied. However, the conditions for persistence from the metapopulation theory deserve much scrutiny in the light of the number of simplifying assumptions made in this study (Giezendanner et al. 2021).

### Catchment models of water-related (WR) disease

Several water-related (WR) diseases show common spreading dynamics, and even similarities in the waterborne ecological cycles for the host(s) or the pathogens. Therefore, the spatially explicit epidemiological framework initiated with epidemic cholera (Bertuzzo et al. 2008; Eisenberg et al. 2013; Rinaldo et al. 2012, 2017) may be extended to a number of other epidemiological contexts of broad hydrological significance. Indeed, the same framework has been employed consistently for describing disease spread in ecological communities for rather different diseases (Rinaldo et al. 2020): epidemic cholera, a neglected debilitating and poverty-reinforcing disease for humans like schistosomiasis, and the high-fatality spread of proliferative kidney disease in salmonid fish. Little impediment exists, therefore, to generalized uses for other diseases within the same framework.

Interest in spatially explicit mathematical models of epidemics has grown considerably in the recent past in a variety of epidemiological contexts (e.g. Rohani et al. 1999; Colizza et al. 2006; Bajardi et al. 2011; Rinaldo et al. 2017; Ciddio et al. 2017; Merler et al. 2015; Chinazzi et al. 2020; Gatto et al. 2020). These models have the potential to account for heterogeneous drivers, and thus to reproduce complex patterns of spreading disease. Current scientific and technological advancements allow us to carry out widespread and relatively straightforward applications of general spatially explicit modeling techniques. Large-scale hydroclimatological and anthropogenic drivers (or their proxies) are key to detailed descriptions of WR disease dynamics, and can often be inferred by remote sensing, satellite imagery and digital image processing, while the tracking of human mobility may be currently done—without any social bias whatsoever even in remote areas—via mobile phones (Wesolowski et al. 2012, 2013, 2014; Finger et al. 2016). Each epidemiological problem needs specific adjustment, however. In fact, many infectious diseases (especially parasitic waterborne ones) share drivers and transmission processes but nonetheless their hosts and/or pathogens are characterized by rather different life cycles, with major implications on the way we infer the mechanisms of the spread of infections (Rinaldo et al. 2020).

Infections caused by viruses, bacteria, protozoans, flatworms, and roundworms are caused by ingestion of, or contact with, water contaminated by specific pathogens. These viral, bacterial, or parasitic infections share similar hydroclimatological and socioeconomic drivers that control pathogen concentrations within the accessible water reservoirs, whatever their nature. It is sad to note that most of the burden of WR infections is attributable to an unsafe water supply, a lack of sanitation, and poor hygienic conditions, which may affect exposure and transmission rates either directly or indirectly. However, the patterns of disease spread are also largely reflected in the spatial heterogeneity of their drivers.

Several climatic drivers, such as rainfall patterns or air and water temperatures, affect transmission rates of contagion. Habitat suitability of pathogens and hosts in waterscapes is controlled by climate, hydrology, and geomorphology concerting the ephemerality of streamflows. epitomized by the relative occurrence of dry-bed conditions (Perez-Saez et al. 2017). This control determines the suitability of host species that are key to the completion of many parasites’ lifecycles and the survival of the pathogen in natural waters. Therefore, hydrology is an essential component of the spatial description of the transmission of WR disease.

For example, a significant test case concerns the prevalence of *Opisthorchis viverrini*, *Schistosoma mekongi* and soil-transmitted helminths (STH), whose public health implications still demand to date maximum attention in a number of countries despite control efforts that include mass-drug administration, and broad education and communication campaigns (Vonghachack et al. 2017). Liver flukes (*Opisthorchis viverrini*), blood flukes (*Schistosoma mekongi*) and soil-transmitted helminths (STH) such as round worm (*Ascaris lumbricoides*), whipworm (*Trichuris trichiura*) and two-hookworm species (*Ancylostoma duodenale*, *Necator americanus*) cause serious infections that often become endemic nationwide but most prevalent in floodplains crossed by major waterways like in the lowlands along the Mekong River, where fish are abundant and local inhabitants prefer to consume traditional dishes prepared with raw fish (Forrer 2012; Vonghachack et al. 2017). Infections with these helminths negatively affect human health and welfare almost worldwide. For example, untreated or chronic infection with *O. viverrini* may lead to severe hepatobiliary morbidity including cholangiocarcinoma, a fatal bile duct cancer. Chronic infection with *S. mekongi* may result in portal hypertension associated with peri-portal liver fibrosis. In certain sites, *O. viverrini* and *S. mekongi* are co-endemic, further increasing the risk of hepatobiliary morbidity (Vonghachack et al. 2017). Finally, anaemia and undernourishment are associated with longlasting infections of these types.

Helminths have complex life cycles, more than those presented for the various macroparasites that cause schistosomiasis. *O. viverrini*, for example, involves two aquatic intermediate hosts, namely freshwater snails (of the genus *Bithynia*) and freshwater fish (of the *Cyprinidae* family). Humans and other mammals are infected by eating raw or undercooked fish. The life cycle of *S. mekongi* involves humans and other mammals (such as dogs, pigs and possibly rats) (Vonghachack et al. 2017). The *Neotricula aperta* snail serves as intermediate host. It lives in the crevices of submerged rocks in the Mekong River (inasmuch as freshwater bryozoans serve as primary hosts for the pathogens of PKD (Carraro et al. 2018a). Even in this case, similarly to the case of schistosomiasis, the *cercariae* emerge from the infected snails during daytime and lie under the water surface. Humans and animals are infected with this parasite via skin penetration when they come into contact with infested waters as in the case of schistosomiasis. Eco-health research studies the prevalence and risk factors of the above macroparasitic infections in humans in the ecological environment where potential animal reservoirs and intermediate hosts, like molluscs and fish, live in close connectivity.

A recent analysis of two population-based models of the transmission dynamics of the worm parasite * O. viverrini* (whose life cycle includes humans, cats and dogs as definitive hosts; and snails and fish as intermediate hosts) includes two models (Buerli et al. 2018). The first model has only one definitive host (humans) while the second model has two additional hosts: the reservoir hosts, cats and dogs. Distributions of the host-specific type-reproduction numbers show that humans are necessary to maintain transmission and can sustain transmission without additional reservoir hosts, suggesting that targeting humans should be sufficient to interrupt transmission of *O. viverrini*. Clearly, a spatially explicit version of the above models is in sight (Rinaldo et al. 2020). A minor but significant modification would stem from a new kind of mobility affecting disease transmission: that of the raw fish, travelling as the catch of the day from fishermen’s communities to nearby markets, that might indeed propagate surviving pathogens.

Other kinds of hydro-epidemiological models emerge, posing interesting novel challenges. For example, the majority of existing models for predicting disease risk in response to climate change are unsuitable for capturing impacts beyond historically observed variability and have limited ability to guide interventions. A recent paper (Beltrame 2018) integrated environmental and epidemiological processes into an interesting ecohydrological model, taking the widespread parasitic disease of fasciolosis as an example. In a nutshell, the liver fluke (*Fasciola hepatica*) life cycle includes ecological interactions with an amphibious mud snail as intermediate host, and two life stages for the parasite involving eggs hatching at suitable temperatures in thin films of moisture, maturing as Myracidia floating or swimming in water actively seeking a snail host. Snails live in muddy area with no (or little) drainage. The model simulates environmental suitability for disease transmission by explicitly linking the parasite life cycle to key weather–water–environment conditions. Using epidemiological data, it was shown that the model can reproduce observed infection patterns in time and space for case studies in UK livestock farming (Beltrame 2018). What is remarkable, in our view, is the hydrologic control exerted by the soil moisture dynamics, linking a different water world. It is interesting that an amphibious mud snail serves as intermediate host in the above example. This suggests another avenue for the study of hydrologic controls on infection disease cycles, i.e. that involving the soil moisture dynamics equation, arguably the most fundamental equation of hydrology (Rodriguez-Iturbe et al. 1999). This could very well be the case for soil-transmitted helminths.

Other cases of soil-moisture controlled disease have been studied, and the relevant hydrology may be significantly different. For example, the incidence of coccidioidomycosis (also called the valley fever, a sometimes lethal respiratory disease caused by a soil-borne fungus, *Coccidioides* spp., whose desiccation under dry spells causes release of airborne spores and their possible inhalation by humans and animals) fluctuates in relation to soil moisture levels from previous summers and falls (Coopersmith et al. 2017). Therefore, one may investigate crossing properties of certain soil moisture levels during antecedent seasons to produce scenarios of infections.

Vector-based diseases are of utmost epidemiological relevance and are in many cases water-related. Mosquito-related disease (like malaria, dengue fever, chikungunya, zika) are water-related because the life cycle of mosquitoes is inextricably linked to ephemeral or permanent water bodies. They have not been dealt with here even though in some cases hydrologic controls exist (Bomblies et al. 2008; Yamana and Eltahir 2013; Whittaker 2019). For example, possible inroads on the control of river blindness (onchocerciasis) may stem from ecohydrologic approaches of the kind studied elsewhere (Rinaldo et al. 2020). Great strides have been made toward onchocerciasis elimination by mass drug administration (Verver 2018) because it is no small endeavour. In fact, an estimated 25 million people are currently infected with onchocerciasis [a parasitic infection caused by the filarial nematode *Onchocerca volvulus* transmitted by the bites of *Simulium* blackflies (Colebunders 2018)]. The transmission dynamics of vector-borne diseases such as river blindness are underpinned by complex interactions between populations of vectors, hosts, and parasites. For onchocerciasis, experimental infections of the insect vector have been conducted to understand and quantify the processes determining vector competence, and indeed the life cycle of the vector suggests the need for very specific hydrologic environments (Colebunders 2018). Their modelling incorporates fundamental mechanistic processes and permits prediction of otherwise elusive epidemiological trends. Standard models of river blindness track the life histories of individual male and female adult blackflies, the vectors, and populations of microfilariae within individual human hosts. The related infection process is usually modeled deterministically, with seasonal variation in transmission defined by monthly biting rates (number of bites per person per month) where age- and sex-dependent heterogeneity in exposure to blackfly bites is considered as well as treatment compliance (Basáñez 2016). Clearly, habitat suitability for blackflies is tied to hydrologic conditions, and thus avenues for mapping the heterogeneity of exposure may be envisioned. As championed at length (Rinaldo et al. 2020), mathematical modelling provides a means to guide the design of interventions targeting these diseases, in terms of their likely effectiveness and cost-effectiveness in attaining control (and possibly elimination) goals (Whittaker 2019). The reader may note that also in this case a spatially explicit epidemiology will find fertile grounds for extensive ecohydrological applications.

## Streamflow ephemerality and disease control

The transmission of waterborne diseases hinges on the interactions between hydrology and ecology of hosts, vectors and parasites, with the long-term absence of water constituting an obvious constraint. The link between spatio-temporal patterns of hydrological ephemerality, transient connectivity and waterborne disease transmission has been investigated in various contexts (e.g. Moir et al. 2004; Tetzlaff et al. 2007; Perez-Saez et al. 2019). The use of limited biophysical and hydroclimate information from otherwise data scarce regions is therefore needed to characterize, classify, and predict river network ephemerality in a spatially explicit context (Perez-Saez et al. 2017). Relevant large-scale ephemerality classification and prediction methodologies have been recently developed, specifically aiming at their epidemiological relevance. They are based on monthly discharge data, water and energy availability, and remote-sensing measures of vegetation. These approaches must maintain a mechanistic link to catchment hydrologic processes to underpin under what conditions the hosts and pathogens can survive.

Far from providing an exhaustive review of this field, here we simply highlight a few relevant examples of applications, in particular on river ephemerality, an important hydrological characteristic affecting waterborne and water-related (WR) diseases owing to its direct and indirect effects at different stages of the transmission cycle. The former include pathogen spread and survival, intermediate host ecology, human exposure to the pathogens, and the contamination pathways of water supply. Indirectly, the changing connectivity of intermittent river networks (per se an important subject of diverse hydrologic research see e.g. Tetzlaff et al. 2007; Garbin et al. 2019; Bertassello et al. 2020a) plays a role in the meta-population dynamics of hosts, vectors, pathogens and humans.

One example that we deem significant for the shown connection of the expansion of the disease range and water resources developments (Steinmann et al. 2006), relates to the ecology of intestinal and uro-genital schistosomiasis caused by *S. mansoni* and *S. haematobium* respectively (Perez-Saez et al. 2017). The disease is contracted during human-water contact through skin penetration of motile free-living larvae, thus exposing local communities during domestic (washing, laundry), leisure (bathing), and livelihood-related activities (fishing, agriculture), with initial contamination occurring as schistosome eggs reach water bodies through faeces or urine. The parasites’ life cycle requires obligatory aquatic snail intermediate hosts, specifically from the genus *Biomphalaria spp.* for the intestinal and *Bulinus spp.* for the uro-genital forms of the disease. The particular significance stems from the environmental economics connections. Schistosomiasis, in fact, is a chronic parasitic waterborne disease that affects an estimated almost a billion people at risk of contracting the disease, of whom roughly 14%) live either in contexts where irrigation schemes have been constructed or in close proximity to large or small dam reservoirs. The linkage between water resources development projects and the spread of schistosomiasis, primarily in sub-Saharan African settings where a stunning 90 % of the world’s burden is concentrated, is undeniable (Steinmann et al. 2006). This is clearly related to the expansion of the range of the obligatory intermediate host for the parasite and by the progressively reduced mean distance of human settlements from the nearest water body—infection occurs through direct contact with water through skin penetration of the parasite. Development and management of water resources matters, therefore, and strategies to mitigate risk factor for schistosomiasis should become integral parts in the planning, implementation, and operation of future water projects. Incidentally, this highlights a tenet of modern economic thinking. In fact, while the impact of improved agriculture is reflected in economic indicators like the GDP of the region implementing the water resources development scheme, the social and economic cost of the increased burden of the disease is hard to predict or quantify. Moreover, schistosomiasis is a poverty-reinforcing, debilitating and neglected disease owing to its low mortality and vast impact only on the poorest layers of the population. Contemporary environmental economics is considering the proper manner to account for the social and economic cost of future loss of workforce, where material and immaterial values should be considered in their own units (say, the number of species rescued from extinction, or the number of averted cases in epidemics, for the latters) (Dasgupta 2013, 2021). We believe that such progress, largely inherent to ecohydrology (2.0) is ready to be used for the betterment of society and in the assessment of the wealth or poverty of Nations.

The connection between ephemeral hydrologic regimes and expansions/contractions schistosomiasis incidence is apparent. Although snails may survive even extended periods of desiccation through aestivation, the specific species matter: *Biomphalaria* snails are much less adapted than *Bulinus* to prolonged dry spells which significantly increase snail mortality. The mean lifespan of *Biomphalaria pfeifferi* is about 40 days, the species within the *Bulinus* genus survive in ephemeral aquatic environments only if they have water for at least two months per year (Perez-Saez et al. 2019). Therefore, hydrologic ephemerality critically determines local habitat suitability for the snail intermediate hosts, in particular for intestinal schistosomiasis, and also conditions human exposure/contamination by limiting the temporal window and the number of locations in which human-water contacts can occur.

The importance of hydrologic ephemerality has already been identified (somewhat separately) by ecologists, social scientists and epidemiologists (Poda et al. 2004, 2006). However, consistent quantitative evaluations implemented on a regular basis are still lacking. A better understanding of the hydrological underpinnings of large-scale ephemerality, including a probabilistic description of the extent of dry channels occurrences that is important towards spatially explicit predictions of schistosomiasis (and by extension of other WR disease) transmission dynamics, with implications for disease control and elimination strategies (Rinaldo et al. 2017). Determinants of stream ephemerality is frequently span multiple spatiotemporal scales across the climatic, vegetation, soil and topographic features (e.g. Perez-Saez et al. 2019). The importance of feedbacks among such features is notable for streamflow generation, but in data-scarce contexts our limited ability to predict the frequency and duration of drybed spells (that is, not simply the probability of zero discharge, but also the much more complex problem of determining the probability of a given number of consecutive days with no streamflow) is a liability. The relative fraction of drybed persistence (a hydroperiod) poses significant methodological challenges. Ephemerality thus proves a component of hydrologic regime classifications whose importance grows progressively in the domain of *ecohydrology 2.0*. Incidentally, hydrological indices derived from streamflow data are quite a few (Bras and Rodriguez-Iturbe 1994; Brutsaert 2015; Hornberger et al. 2014), including exact results like e.g. the determination of the atom of probability at the origin of ephemeral streamflow duration curves (e.g. Botter et al. 2010) or the frequency of 0-discharge periods (Botter et al. 2007). In this domain the classification of ungauged rivers today mostly relies on remotely-sensed and objectively manipulated catchment characteristics, such as contributing area and slope (e.g. Band 1986; Tarboton 1997), and climatic information, such as precipitation and temperature (e.g. Borga 2002; Anagnostou et al. 2004; Michaelides et al. 2009; Mirabbasi et al. 2013).

Exploiting hydrologic controls to sharpen our knowledge of the habitat suitability for hosts/pathogens of WR disease is a lively research field. For example, current strategies to interrupt schistosomiasis transmission target the freshwater snails that are the necessary intermediate host of schistosome parasites, like e.g. the effects of prawn aquaculture on poverty alleviation and schistosomiasis control (Hoover et al. 2019). Moreover, predictors of snail presence/abundance tend to be more stable in space and time than snail habitat suitability models (Perez-Saez et al. 2019), and nonetheless may still utilize remotely acquired and objectively manipulated environmental proxies (like e.g. the relative fraction of water surface area covered by floating vegetation). Unlike snail surveys, proxies are easily estimated from drone or satellite imagery (Wood et al. 2019; Rabone et al. 2019).

To achieve elimination of chronic disease like schistosomiasis, however, more sensitive diagnostic tools may be needed to monitor the transmission progress, or else its interruption in the environment, especially in low-intensity infection areas. One promising avenue concerns the development of eDNA-based tools. For instance, a recent study (Sengupta et al. 2019) pinpointed the importance to efficiently detect DNA traces of the parasite *Schistosoma mansoni* directly in the aquatic environment (Rinaldo et al. 2020). Remarkably, the study has achieved a comparative analysis of the detectability of ‘true’ eDNA at very low concentrations of *cercariae*, and tested the field applicability of the method at known transmission sites by comparison of schistosome detection by conventional surveys (snail collection and cercariae shedding) with eDNA in water samples (Sengupta et al. 2019). The substantial agreement between the methods when surveys provide a baseline and, critically, the detection of schistosome presence at sites where snail surveys failed, suggest that eDNA will likely provide in the future a key tool also for field epidemiology of WR disease.

Ephemerality may affect biological invasions as well, a subject surely relevant to ecohydrology, are treated elsewhere (Kolmogorov et al. 1937; Elton 1958; Campos and Mendez 2005; Campos et al. 2006; Méndez et al. 2003, 2004; Bertuzzo et al. 2007; Méndez et al. 2010; Rinaldo et al. 2020). Suffice here to mention the intertwined dependences on hydrologic controls that involve species-specific ecological traits with the formation of hubs of connectivity and habitat suitability (Morel-Journel et al. 2019). Examples of the interactions between the features of the invaded landscape and the internal dynamics of an introduced population [e.g. due to the experimental introductions of *Trichogramma chilonis* (Hymenoptera) in artificial laboratory microcosms] proved notable to that end, showing that the spread is affected by clusters, hubs and small-population mechanisms such as Allee effects (Morel-Journel et al. 2019). Similar results have been obtained in the laboratory with protists (e.g. Holyoak and Lawler 2005; Carrara et al. 2012, 2014; Giometto et al. 2015, 2017) or yeast (e.g. Giometto et al. 2018).

### Scaling of carbon cycling and fluvial corridors: global issues

Natural ecosystems often exhibit deep structural similarities emerging across scales of space, time and organizational complexity (Levin 1992). Scale invariance of ecological patterns offers a powerful tool to make way for coherent, unified descriptions, especially in river networks whose geometry, topology and geomorphology affect their role as ecologic substrates (Rodriguez-Iturbe and Rinaldo 2001). If the part and the whole of the substrate for interactions are statistically indistinguishable across many orders of magnitude, implications abound for the function of the embedded biota, as shown in detail elsewhere (Rinaldo et al. 2020). Several areas where results are still missing do exist, however. One deals with scaling of carbon storage in inland waters, recently argued to play a central role in the global carbon cycle (Battin et al. 2009).

Metabolic principles of river basin organization have been, and will be, well sought (e.g. Rodriguez-Iturbe et al. 2011). If we define the metabolism of a river basin as the set of processes through which the basin maintains its structure and responds to its environment, then several links with hydrology at large scales exist—meaning not simply the effects of a single organism, but rather those of a large and diverse assemblage of organisms like vegetation at catchment scales. Green (or biotic) metabolism is measured by transpiration and blue (or abiotic) metabolism through runoff, with distinctions arising when sinks in biofilms prove significant (Battin et al. 2009). In fact, currently the “boundless carbon cycle” must be seen at a different angle, as the terrestrial biosphere is assumed to take up most of the carbon on land, but it is now clear that inland waters process large amounts of organic carbon (Battin et al. 2009; Horgby et al. 2019). Approximately 0.6 Pg C/yr are buried in inland water sediments, approximately equivalent to 20% of the carbon assumed to be uptaken by terrestrial biomass and soils. Still, these estimates do not include long-term net carbon burial in floodplains and other near-water landscapes that probably make for another significant flux (Battin et al. 2009). It has been argued convincingly that the loss of organic carbon from terrestrial ecosystems and its subsequent burial in inland waters represents a redistribution of carbon sinks that must be taken into account in climate change mitigation strategies (Battin et al. 2009).

Other questions come to mind. Allometric scaling of metabolic rates with organismic size has been variously addressed in the biological literature and network theory under the banner of Kleiber’s law (Kleiber 1947). Decades of research in field and theoretical ecology have unraveled a wide array of ecological scaling laws pertaining to the distribution of species, their abundances and metabolic requirements (Brown et al. 2004). These laws hold for most ecosystems on Earth and help predict how ecological communities assemble in the environment. Relatedly, physiological studies have shown that metabolic rates, *B*, scale with body mass, *M*, according to a universal power law, $B = c M^\alpha$ (where $\alpha$ is a scaling exponent, and *c* a constant). This is Kleiber’s law (KL) (Kleiber 1947; Calder 1984; McMahon and Bonner 1983), which has been claimed to holds across more than 27 orders of magnitude of body mass—from molecules of the genetic code and metabolic machinery to whales and sequoias (West and Brown 2004). The metabolic power required to support life across that range spans over 21 orders of magnitude and is fundamental for our understanding of ecology across scales. It is widely accepted that KL applies across species with $\alpha = 3/4$ in many groups of organisms, a predominance termed “the central paradigm of comparative physiology” (Brown et al. 2004). Metabolic demand per unit mass thus decreases as body mass increases. For higher organisms, KL reflects both the ability of the organism’s transport system to deliver metabolites to the tissues and the rate at which the tissues use them. The universality of the exponent 3/4, however, has been challenged by robust empirical evidence of a wealth of exponents $\alpha$ deviating from 3/4 and by arguments suggesting alterations of the power law relation. Implications of the structure of KL are claimed to include many fundamental processes, such as life history attributes (including development rates, mortality rate, age at maturity, lifespan, and population growth rate), population dynamics and interactions (including carrying capacity, strength of competition and predation, and patterns of species diversity), and ecosystem processes (including rates of biomass production and respiration). This led to a metabolic theory of ecology of broad aims (Brown et al. 2004) and to discussions of its limits and validity (e.g. Zaoli et al. 2019).

All this matters to metabolic principles of river network organization. In fact, the above results suggest by analogy a possible relation of overall metabolic rates to total cumulative area at any point of a river network (Banavar et al. 1999). In fact, in Euclidean geometry a *D*-dimensional object, a 3D body or an almost 2D catchment, is characterized by a linear size *L* and a “body” size [i.e. mass *M*] that scale as $M \propto L^D$. To model the metabolic system of living organisms, it has been postulated that the fundamental processes of nutrient transfer at the microscopic level are independent of organism size. In a D-dimensional organism, the number of such transfer sites scales as $L^D$ (Banavar et al. 1999). Each transfer site is fed with nutrients (for example, through blood) by a central source through a network providing a route for the transport of the nutrients to the sites. The total amount of nutrients being delivered to the sites per unit time is the equivalent of the metabolic rate *B*, and simply scales as the number of sites or $B \propto L^D$. The total blood volume, proportional to the body size *M*, depends, in steady-state of supply and demand of metabolites, on the structure of the transportation network. It is proportional to the sum of individual flow rates in the links or bonds that constitute the network. In a very broad class of efficient or simply directed networks one has $M \propto L^{D+1}$ (Banavar et al. 1999). Thus the total blood volume increases faster than the metabolic rate *B* as the characteristic size scale of the organism increases. Thus larger organisms have a lower number of transfer sites (and hence *B*) per unit blood volume. Metabolic rates do not scale linearly with mass, but rather as $B \propto M^{D/(D+1)}$, or $\alpha = D/(D+1)$ (Banavar et al. 1999), a result suggesting the fully 3D bodies should have $\alpha =3/4$ and flat 2D organisms $\alpha =2/3$.

For a river network, delivering water and sediments to maintain its function, one therefore argues that $D=2$ and $\alpha \sim 2/3$. To test this, it has been shown (Maritan et al. 2002) that total contributing area $A_i$ at any site *i* plays the role of the basin metabolic rate $B_i$, whereas the analog of organismic mass at *i* , $M_i$, is defined by the quantity $M_i \propto \sum _{j \in \gamma (i)} A_j$, where $\gamma (i)$ indexes all paths connected to *i*, i.e. the integral of all fluxes in steady state. The main result (Maritan et al. 2002) is that indeed from vast empirical testing one retrieves $\alpha = 2/3$ with minimal scatter. Evidence also suggests that ensemble averaging of the allometric property (where individual realizations are different networks) leads to results in excellent accord with the limit scaling of efficient networks. Network-related allometric scaling in living organisms is thus regulated by metabolic supply-demand balance where scaling features are robust to geometrical fluctuations of network properties.

The related research questions are deeply connected with the tenet of ecohydrology. Are there meaningful principles of equal metabolic rate per unit area throughout the basin structure? Such principles would have profound implications on large-scale geochemical cycles, and they seem likely because of the very spatial organization of river basin hydrologic dynamics. Do we have a definitive view on whether the basin structure itself leads to a power law for the probability distribution of metabolic rates, like e.g. transpiration from a randomly chosen subbasin? Empirical evidence has merely suggested that river basin metabolic activity is linked with the spatial organization that takes place around the drainage network and therefore with the mechanisms responsible for the fractal geometry of the network (Rodriguez-Iturbe et al. 2011). A new coevolutionary framework for biological, geomorphological, and hydrologic dynamics may very well be in sight.

Streams and rivers play a major role in the global carbon cycle because they collect, transform and deliver terrestrial organic carbon to the oceans. The rate of dissolved organic carbon (DOC) removal depends on hydrological factors, primarily water depth and residence time. These factors change quite predictably within a river network, that assumes universal scaling geomorphic features regardless of size, vegetation, exposed lithology or climate (Rodriguez-Iturbe and Rinaldo 2001). Therefore, local DOC concentration and composition is the result of transformation and removal processes occurring in the whole upstream catchment. The nonlocal character of any local interactions, whether of physical, chemical or biological nature, is subsumed by the master variable $A_i$, total contributing area at any point *i* in the river network.

Recently, theory of the form and scaling of river networks was combined with models (Bertuzzo et al. 2018) and experiments (Wollheim et al. 2015) of DOC removal from streamwater to investigate how the structure of river networks and the related hydrological drivers control DOC dynamics. Therein, it was found that the physical process that shapes the topological and metric properties of river networks, imperfect minimization of total energy dissipation leading to dynamically accessible states leads to structures that are also more efficient in terms of total DOC removal per unit of streambed area. This unintended consequence of nature’s operation echoes the increase of metapopulation capacity with evolving networks.

**Fig. 4:**
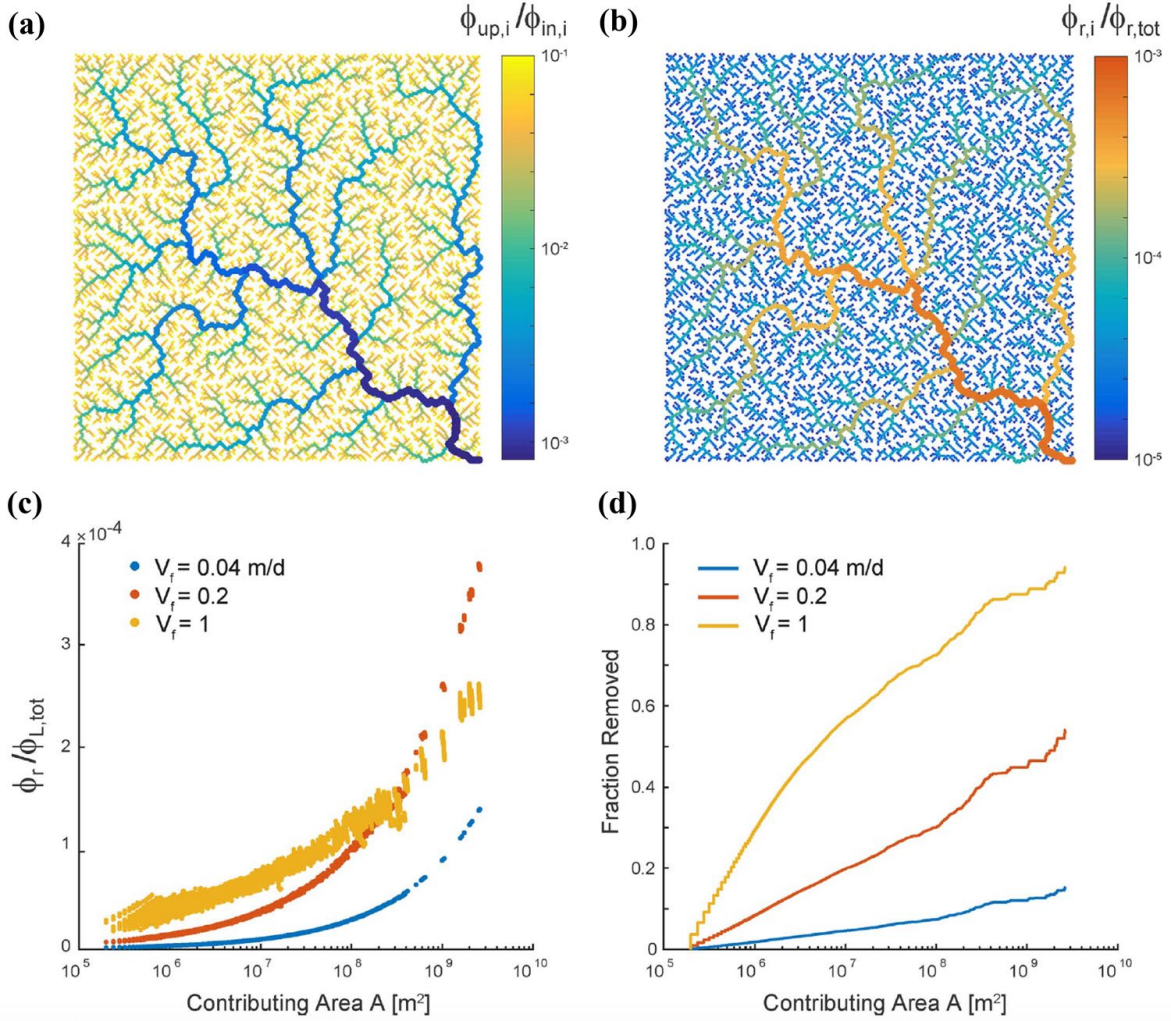
Distribution of DOC removal along optimal channel networks (OCNs). Panel **a** and **b** show the OCNs used for the simulations. Color codes represent: **a** the fraction of mass input to each reach that is removed by that same reach and **b** the contribution of each reach to the total network-scale removal. Note that the removal flux of each reach is normalized by the total load as a function of the master variable for stream ecology, total contributing area *A* at any reach. In this sense the scaling of carbon removal is directly related to he scaling aggregation features of the optimal channel network, in analogy of the scaling of metapopulation capacities (Fig. 2). For every reach of the river network, the downstream mass flux can be expressed as a function of the input flux, and removal as a faction of the total. A well-known feature is that average flow velocity increases (mildly) downstream (Rodriguez-Iturbe and Rinaldo 2001), thus reducing residence time for equal reach lengths. Therefore, hydrological and geomorphological drivers can exert a critical control on elemental removal at the river network scale; **c** removal flux of each reach, $\phi _{r,i}$, normalized by the total load, termed $\phi _{L,tot}$, as a function of the master variable for stream ecology, total contributing area *A* at any reach. **d** Fraction of the total load that is removed by streams with contributing area smaller than *A*. Top panels show results for an uptake velocity $V_f = 0.2$ [m/d] (the latter is defined as the ratio between the removal of a given element per unit of streambed surface area [M/L$^2$ T] and its concentration in the water column [M/L$^3$]. The model thus assumes that most of the processing occurs at water-sediment (benthic biota) or water-air (i.e. photodegradation) interfaces). For every reach *i* of the river network, the downstream mass flux $\phi _{r,i}$ [M/T] can be expressed as a function of input flux, $\phi _{in,i}$ and removal via $\phi _{r,i} = \phi _{in,i} \exp [-V_f \tau _i/d_i]$ where $d_i$ is the depth of the water column and $\tau _i=w_i d_i L_i/ Q_i$ is the residence time of water within reach *i* and $\tau _i = w_i d_i L_i /Q_i$ is the travel time of water within reach *i*. $V_f$ is a biological measure independent of hydrological conditions because it is based on per unit area removal and is well suited for comparing biological activity in streams of different sizes (Bertuzzo et al. 2018). Thus, even at constant $V_f$, the removal rate decreases downstream as water depth increases. Therefore, hydrological and geomorphological drivers can exert a critical control on elemental removal at the river network scale. Other parameters are reported in the reference article, after (Bertuzzo et al. 2018; Helton et al. 2018) (color figure online)

The structure of river networks also induces a scaling of the DOC mass flux with the contributing area that does not depend on the particular network used for the simulation. This fact, that proves totally robust to spatial heterogeneity of model parameters, echoes another property of river networks as ecological corridors, the inevitability of the topology seen by the backbone for propagation of a biological invasion along a network that explained patterns of human migrations (Rinaldo et al. 2020). In this case, it was found that such scaling enables the derivation of removal patterns across a river network in terms of clearly identified biological, hydrological and geomorphological factors (Bertuzzo et al. 2018). In particular, the fraction of terrestrial DOC load removed by the river network scales with the catchment area *A* (and with the area of a region drained by multiple river networks). This results proves of particular importance (Fig. 4).

It should be acknowledged that a direct comparison of scaling relationships with empirical data could be hindered by the fact that the framework in Fig. 4 focuses on the terrestrial deliveries of DOC and ignores instream DOC sources (Bertuzzo et al. 2018). The release of DOC from algae and microorganisms can greatly contribute to the overall DOC pool in streams during baseflow. Therefore, a sensible conclusion is that this autochthonous DOC is generally highly available to heterotrophic metabolism, and is therefore thought to yield high uptake velocities. This labile DOC could easily facilitate the removal of terrestrial DOC, an interaction called ‘priming’ (Hotchkiss and Hall 2015). Therefore, future modeling efforts should focus on combining the various carbon sources intrinsically diverging in bioavailability and hence in uptake velocities (Bertuzzo et al. 2018). The scaling relationships shown in Fig. 4 (Bertuzzo et al. 2018) characterizes basal patterns of terrestrial DOC, the main source of organic carbon in most streams, and deviations from its attainment may indicate the relevance of autochthonous DOC generation (Bertuzzo et al. 2018).

Previous efforts to characterize the role of network structure and stream size on carbon and nutrient removal relied on the concepts of stream order and Horton’s ratios (Raymond et al. 2016). It has been noted (Bertuzzo et al. 2018; Helton et al. 2018) that a significant advantage of a framework that works with fully realistic metrics (e.g. individual reach lengths and widths, and total contributing area at any site) is to underpin distinctive descriptions of different trees. The structure of river networks cannot be uniquely defined by inevitable features like those emerging from average metric properties or topological indices defined by stream orders or Horton ratios that iron out details and distinctiveness (Kirchner 1993; Rinaldo et al. 1999; Rodriguez-Iturbe and Rinaldo 2001). Besides, the computation of mean stream length via Strahler’s ordering is tricky, and may lead to severely overestimated mean network lengths (Rinaldo et al. 1999). The availability and the analysis of digital elevation models of the Earth’s surface has rendered the use of concepts like stream order obsolete (Rodriguez-Iturbe and Rinaldo 2001). Indeed, contributing area proved the key variable to describe the scaling of DOC removal patterns across river networks.

Scaling studies have furthered our understanding of carbon sinks in inland waters and opened new areas for investigation. Low-hanging fruits are relaxations of simplistic assumptions, like those of steady-state hydrology. The major implication of those studies is that the impact of instream processes on carbon cycling can be clearly related to the global scales—once freed of previous untenable simplifications.

## eDNA, species dispersal and river networks

Environmental DNA (eDNA), present as loose fragments, shed cells or microscopic organisms, can be extracted from their transport matrix, whether water or soil (see Tsuji et al. 2019 for a review of methods for collection, extraction and detection, and Bass et al. 2015 for the powerful techniques currently available in molecular biology). Its identification, now to be considered routine, may be used to track the presence of target species. With the ever-increasing technical proficiency in the biological laboratories we are currently capable of mapping even the composition of entire communities, and the extended use of eDNA analyses provides a rich new source of spatial and temporal data allowing us to address research questions that have so far defied empirical verification (Bálint et al. 2018). Approaches using eDNA for qualitative species detection have already proved their worth for species management and biodiversity conservation, by improving substantially field evidence in a replicated and consistent manner and by underpinning the detection of rare invasive or parasitic species (Carraro et al. 2018b; Rinaldo et al. 2020).

eDNA measurements within catchments, especially in river waters, provide a snapshot record of the species present within the control volume—usually upstream of the sampling port if transported by hydrologic waters—but also that the interpretation of its signals is a rather complex issue affected by significant uncertainties (Carraro et al. 2018b, 2020a, 2021a). A key process that allows us to underpin spatial patterns of its sources relates to the fact that, once released to the environment, eDNA undergoes selective decay. Nucleic acids incur progressive damage during, say, hydrological advection, retention and resuspension (Shogren et al. 2017) resulting in alterations that affect eDNA detection in whatever environmental sample—which in turn involves a role for hydrologic controls. Most importantly, eDNA has polydisperse properties due to its origin from diverse organic sources (e.g. spores, cells, tissues, faeces), which complicates the evaluation of decay rates and the analysis of the possible source—in a truly remarkable inverse problem examined (Rinaldo et al. 2020). As discussed therein, the eDNA sampled at any point within a dendritic network of sources is the outcome of diffuse eDNA release from many possible source points upstream, modified after emission by decay processes during their transport. The diverse pathways to the sampling site are governed by network connectivity, in which each path to the observation point may be described by different hydro-morphological conditions. As a result, while it is relatively straightforward to link a positive test with the presence of the target species at some (unknown) distance upstream, quantification of species densities and the location of populations is currently impossible because, besides a number of potentially confounding factors affecting eDNA shedding [e.g. animal behavior, movement, physiology and size (Carraro et al. 2018b)], it requires consideration of the effects of the dynamics of eDNA transport along river branches and the deconvolution of the hierarchical aggregation of the various network branches. In part, the latter gap has been recent filled with reference to the pathogen of proliferative kidney disease (PKD) in freshwater fish (Carraro et al. 2016, 2017, 2018a, b).

**Fig. 5:**
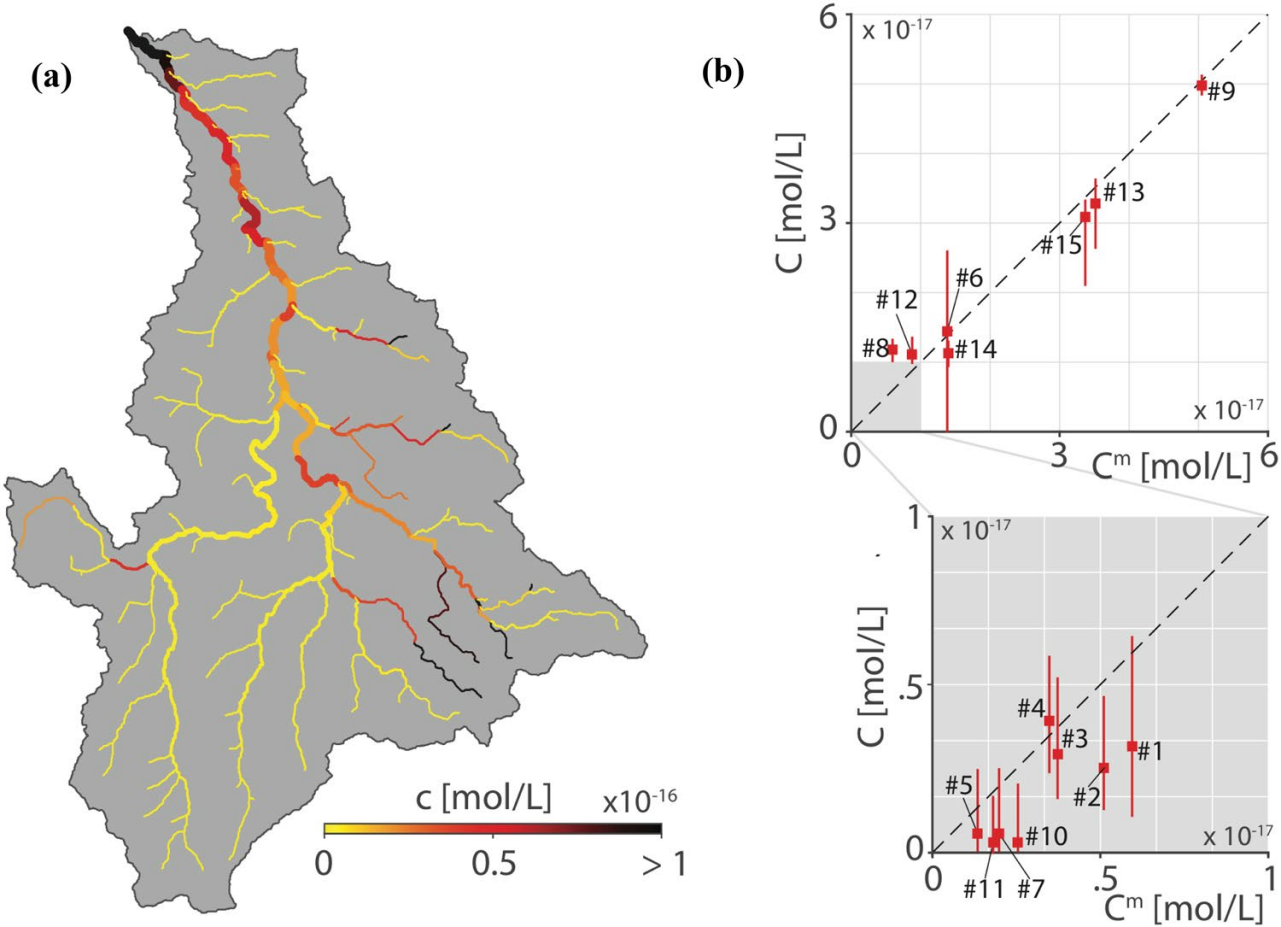
eDNA results allow to trace the spatial location of the bryozoan *Fredericella Sultana* to tackle the epidemiological and demographic dynamics of the deadly Proliferative Kidney Disease (PKD) hosts (bryozoans and fish) (Carraro et al. 2018a). **a** Map of local eDNA concentrations obtained from averaging results from habitat suitability models (Carraro et al. 2018a). **b** Modelled (*C*) vs. observed ($C^m$) *F. sultana* eDNA concentration. Red lines identify 10th–90th percentile ranges of the distribution of all accepted models; squares represent values averaged over all accepted models. (inset) Zoom from panel **b** (after Carraro et al. 2017, 2018b)

One significant example is shown in Fig. 5. It relates to tests performed by contrasting joint field measurements of eDNA concentrations of the myxozoan parasite *Tetracapsuloides bryosalmonae* and its primary host, the freshwater bryozoan *Fredericella sultana*, across various locations within the Wigger watershed (Switzerland) (Carraro et al. 2018b). *T. bryosalmonae* is the causative agent of Proliferative Kidney Disease (PKD), a high-mortality disease affecting salmonid fish populations (Rinaldo et al. 2020). PKD is acknowledged as a leading cause of the decline in brown trout populations in Europe. It affects diverse salmonid populations in North America and is a major aquaculture pathogen (Burkhardt-Holm et al. 2005; Okamura et al. 2011). The water samples used for eDNA detection of both *F. sultana* and *T. bryosalmonae* were collected at regular intervals at 15 sites during 12 months (one 500-mL sample per sampling occasion and site). *T. bryosalmonae* eDNA is likely to be largely derived from spores shed into the environment. Parasite spores, released into water by infected bryozoans, infect brown trout through skin and gills and proliferate in the kidney. To complete the life cycle, spores infective to bryozoans are excreted in the urine of infected fish. These two types of spores are genetically indistinguishable yet differentiated in terms of function, which poses further challenges for modelling. The *T. bryosalmonae* eDNA concentration may thus be a product of the genomic contents of the two types of spore originating from very different transport sources, i.e. from an immobile source (bryozoans) coupled with a mobile source (fish). In this case, a comparative analysis of field-measured eDNA for both *F. sultana* (sessile source of eDNA), and *T. bryosalmonae* (eDNA that could jointly originate from sessile and mobile hosts) proves particularly instructive as a demonstration of the potential of the framework proposed in Carraro et al. (2018b).

The example briefly described here is of interest because it proved capable of interpreting both eDNA data incidentally shed from benthic populations (*F. sultana*, with likely sources of eDNA from faecal pellets and sloughed cells) and eDNA from spores released into the water (*T. bryosalmonae*) (Carraro et al. 2018b). Inherent complexities are not ignored. For instance, although different forms of eDNA may be differently impacted by environmental factors such as temperature and pH, the choice of formulation involving a single parameter expressing the decay time for both species appeared satisfactory for capturing the integrated eDNA transport dynamics at the catchment scale.

Accurate field validations of the current assumptions are needed to generalize this framework, and this could be achieved by relatively simple experimental designs. For instance, the displacement and decay of genetic material from non-native known biomasses placed in well differentiated positions (say, within a catchment where hydrologic and geomorphologic drivers are known) could be a key factor (Laporte et al. 2020). Subsequent sampling at downstream sites, where eDNA would be contributed by sources at known distances, could then be used to assess the strengths and weaknesses of each assumption underlying the proposed approach (Carraro et al. 2021b).

Tracking the source area and the local biomass density of target species via downstream eDNA measurement is therefore within reach, provided that a suitable spatially explicit framework is used to interpret the field data. Key is accounting for the type of filtering produced by the progressive damage occurring during hydrological transport, and harnessing it to recover spatial information on species distributions. The integration of quantitative eDNA measurements, hydro-geomorphological scaling and ecological models presented here has opened yet a novel direction in ecohydrological studies by unlocking the huge potential of remote monitoring using eDNA.

## Conclusion

We conclude that time is ripe for a community redefinition of the domain of ecohydrology. The hydrological and ecological communities should acknowledge the fundamental unity of materials and methods, and the broad reach, that bind together the studies of hydrologic controls on the biota where catchments act as the fundamental control volume.
